# A machine learning algorithm for the detection of paroxysmal nocturnal haemoglobinuria (PNH) in UK primary care electronic health records

**DOI:** 10.1186/s13023-024-03406-4

**Published:** 2024-10-13

**Authors:** Amanda Worker, Hadley Mahon, Jack Sams, Freya Boardman-Pretty, Elena Marchini, Rand Dubis, Alan Warren, Jez Stockdale, Jyothika Kumar, Elizabeth Varones, Daniel Ollerenshaw, Calum Grant, Peter Fish, Richard J. Kelly

**Affiliations:** 1 Mendelian, The Trampery, Old Street, London, UK; 2 St. James’s University Hospital, Leeds, UK

**Keywords:** Paroxysmal Nocturnal Haemoglobinuria (PNH), Machine learning, Rare disease, UK electronic health records, Primary care

## Abstract

**Background:**

Paroxysmal Nocturnal Haemoglobinuria (PNH) is an ultra-rare, acquired disorder that is challenging to diagnose due to varied symptoms, heterogeneous patient presentations, and lack of awareness among healthcare professionals. This leads to frequent misdiagnosis and delays in diagnosis. This study evaluated the feasibility of a machine learning model to identify undiagnosed PNH patients using structured electronic health records.

**Methods:**

The study used data from the Optimum Patient Care Research Database, which contains electronic health records from general practitioner (GP) practices across the United Kingdom. PNH patients were identified by the presence, and control patients by the absence of a PNH diagnosis code in their records. Clinical features (symptoms, diagnoses, healthcare utilisation) from 131 patients in the PNH group and 593,838 patients in the control group, were inputted to a tree-based XGBoost machine learning model to classify patients as either “positive” or “negative” for PNH suspicion. The algorithm was finalised after additional exclusions and inclusions applied. Performance was assessed using positive predictive value (PPV), recall and specificity. As the sample used to develop the algorithm was not representative of the true population prevalence, PPV was additionally adjusted to reflect performance in the wider population.

**Results:**

Of all the patients in the PNH group, 27% were classified as positive (recall). 99.99% of the control group were classified as negative (specificity). Of all the patients classified as positive, 60.4% had a diagnosis of PNH in their record (PPV). The PPV adjusted for the population prevalence of PNH was 19.59 suggesting nearly 1 in 5 patients flagged may warrant further PNH investigation. The key clinical features in the model were aplastic anaemia, pancytopenia, haemolytic anaemia, myelodysplastic syndrome, and Budd-Chiari syndrome.

**Conclusion:**

This is the first study to combine clinical understanding of PNH with machine learning, demonstrating the ability to discriminate between PNH and control patients in retrospective electronic health records. With further investigation and validation, this algorithm could be deployed on live health data, potentially leading to earlier diagnosis for patients who currently experience long diagnostic delays or remain undiagnosed.

**Supplementary Information:**

The online version contains supplementary material available at 10.1186/s13023-024-03406-4.

## Background

Paroxysmal nocturnal haemoglobinuria (PNH) is an acquired clonal haematopoietic disorder. It is a rare disease with a prevalence of 3.81 per 100,000 [1] and is characterised by anaemia, severe intravascular haemolysis, renal dysfunction, bone marrow failure and frequently, life threatening thrombosis. Presentation in primary care may include more generalised and early symptoms such as fatigue, shortness of breath, haemoglobinuria and impotence in men.

While the United Kingdom (UK) is uniquely positioned to diagnose and treat PNH patients within the PNH National Service [2], and treatment is available [3, 4], access primarily depends on the recognition of symptoms in primary or secondary care settings. This can be challenging for a number of reasons; (1) lack of knowledge and understanding of this ultra rare disease, (2) symptom overlap with other conditions that may be more common or well known, and (3) heterogeneity in individual symptom profiles making it more difficult for even knowledgeable practitioners to recognise [5]. All of these factors combined contribute to a diagnostic delay where roughly 35% of PNH patients experience symptoms for 12 months or longer before receiving a correct diagnosis [4]. Furthermore, around 13% of PNH patients have received an incorrect diagnosis and in some cases inappropriate treatment programmes before receiving the correct diagnosis of PNH [4].

Primary care data in the UK has been increasingly recorded electronically over the past several decades, containing both structured (codes such as Read V2 and V3/CTV3 or SNOMED-CT) and unstructured data (note taking, discharge letters) [6]. The structured data alone are an extremely rich source of information containing information on GP visits, symptoms, diagnoses, referrals and test results. In recent years, some structured data has become available for research and development purposes [7]. This provides a unique opportunity to learn about the medical history of patients and to build tools which may aid in “case-finding”. “Case-finding” refers to flagging patients who meet criteria to be tested for a specific disease, but do not have the diagnosis coded in their electronic health record, thus indicating a potentially undiagnosed case.

Case-finding algorithms can be developed using various approaches. A simple approach might involve identifying signs, symptoms, and diagnostic criteria from the literature and then searching electronic health records for matching profiles. However, the challenge with PNH lies in its wide range of symptoms, often overlapping with other conditions, and the existence of diverse clinical phenotypes [8]. Such complexities necessitate a more sophisticated approach. Machine learning algorithms offer a solution by "training" on real-world PNH patient profiles. This allows the model to learn subtle patterns and combinations of features that may not be detected with simpler rules-based approaches. By testing the algorithm's learnings on unseen patient data, we can evaluate its ability to accurately identify potential PNH patients.

This study aimed to evaluate the feasibility of developing a machine learning model to identify potentially undiagnosed PNH patients using data from the Optimum Patient Care Research Database [7]. We built and evaluated a novel algorithm on two groups of patients: a PNH group with a PNH diagnosis coded in their record and a control group without a PNH diagnosis coded. This evaluation sought to assess to what extent the algorithm could identify the PNH group vs the group without a PNH diagnosis. The future objective of this work is to translate this research-based algorithm into a clinical case-finding tool that can be applied to real-world, live health data to identify undiagnosed PNH patients, potentially leading to earlier diagnosis and treatment.

## Methods

### Data source

This study used the Optimum Patient Care Research Database (OPCRD) [7], which at the time of development consisted of 23 million de-identified patient records from patients registered at GP practices across the UK. The data fields available in OPCRD include demographics, clinical events including diagnoses and symptoms and measurements such as blood test results, (coded using SNOMED and CTV3/Read codes) [9], referrals and prescriptions.

### Data sets

A study data set was constructed consisting of a PNH group and a control group (non-PNH patients). Patients were determined by the presence of a diagnostic SNOMED-CT code ('1963002') for PNH in the electronic health record while controls were determined by the absence of a diagnosis code for PNH. Out of the total dataset, there were 186 unique patient IDs that included a code for PNH, and all were selected to be included in the PNH group. Of all the patients in OPCRD with no diagnosis code for PNH present, 700,000 were randomly selected as controls in this study.

### Data cleaning and preprocessing

Data extraction was carried out using Structured Query Language (SQL) to retrieve essential patient details (patient ID, practice ID, year of birth, sex) and encompassed clinical events spanning the entire patient record.

Prior to any transformative or statistical analyses, cleaning and preprocessing procedures were implemented on the dataset. In instances where a SNOMED-CT code was absent but a Clinical Terms Version 3 (CTV3) read code was present, a mapping process was undertaken. The CTV3 read code was associated with the relevant SNOMED-CT code, which was then used to fill in missing fields. In specific cases, the SNOMED-CT code column contained Description ID values. To ensure the accurate identification of relevant SNOMED-CT codes, these Description IDs were substituted with their corresponding mapped SNOMED-CT codes.

Mapping files for CTV3 read codes, Description IDs, and SNOMED codes were generated from SNOMED CT UK Edition reference files obtained from National Health Service Technology Reference Update Distribution (NHS TRUD) [9].

Events were identified as duplicated within the same subject if the patient ID, event date, CTV3 read code, SNOMED-CT code, and practice ID were identical. In such instances, only the initial occurrence was retained, or in cases involving numeric values (fields: 'numeric_1' and 'numeric_2'), the instance containing numeric values was preserved.

Given the availability of only birth year in OPCRD, a standardised birth date (July 1st + year) was used for age calculations. This standardisation was essential for computing the patient's age at the time of each recorded event, with a potential age deviation of no more than six months. Records lacking any recorded events or birth year information were excluded from subsequent analyses.

Partial and full duplicated records is an ongoing issue in OPCRD. For example, if a patient has moved practice multiple times, parts of their electronic health record may have been recorded multiple times under a different patient ID. This is problematic for statistical and machine learning analysis, as (a) partial records reduce the power in the data if some relevant clinical features are not recorded for the correct patient ID, and (b) duplicated records (i.e. the same patient with a different patient ID) could end up in both the PNH group and control datasets if the PNH diagnosis has not been recorded in one of those instances. This makes it more challenging to learn about specific and relevant features to the disease. In some instances it was possible to use birth year, PNH diagnosis date and the events recorded to merge and deduplicate patients.

In the PNH group, all clinical events up to the date of the diagnosis code were analysed for the study; if the date of diagnosis was missing the record was excluded from further analysis. For controls, a random index date was assigned, from any date between the first and last recorded events, to better match the partial records of PNH patients caused by the pre-diagnosis cut-off; all clinical events up to that point were analysed. PNH patients and controls were not matched based on any features in the model and thus the results are indicative of performance in the general population.

### Feature and code selection

In machine learning, a feature is a measurable characteristic or piece of information used by the model to make decisions. Features can be quantified and serve as inputs for the model to learn patterns and relationships within the data. Features included in the model fall into three categories: (1) clinical features which included symptoms and conditions associated with PNH (e.g. thrombosis), (2) healthcare utilisation (e.g. urology referrals, number of blood tests), (3) exclusionary symptoms and procedures that make it highly unlikely that PNH is present (e.g. bone marrow transplant). The features were selected based on the literature [3–5, 7, 10, 11], in consultation with a leading Consultant Haematologist at the Leeds National PNH centre and a review of case reports and case electronic health records (see Supplementary Table 1 for features and basis of selection). Review of case reports involved scanning through “Real Stories” published online [12], to identify additional features that may increase the power in the primary care data for classification purposes. Review of the PNH patient electronic health records was conducted after data cleaning and involved scanning the clinical events to identify features that commonly occur in PNH patients but may not be considered clinically indicative of PNH, such as skin infections.

In rare disease research, sparsity of primary care data can present significant challenges with algorithmic approaches. This sparsity occurs for several reasons including, (1) extensive SNOMED-CT code lists for the same or very similar conditions (e.g. several codes exist for abdominal pain) such that if all codes are not included, all instances of abdominal pain are unlikely to be identified in the data, (2) different coding working practices, both across different healthcare practices/GPs and over time (electronic health coding increases from the year 2002 onwards), and (3) heterogeneity in clinical presentation of the disease. A SNOMED-CT code list was created for each feature. Code lists were designed to be inclusive and to capture the nuances of SNOMED-CT coding to reduce code related sparsity. For example, we included one clinical feature named “kidney dysfunction” which combined codes related to chronic kidney disease, renal insufficiency and acute kidney injury.

### Data transformation

Using the SNOMED-CT code lists defined for each feature, the cleaned clinical events table was searched for the relevant codes and transformed into a quantitative dataset. Features were in two categories: (1) the normalised count of occurrences (number of occurrences across the record, divided by the length of the electronic health record) and (2) the age of onset (age the patient was when the feature first appeared).

### Final sample and feature selection

**Fig. 1:**
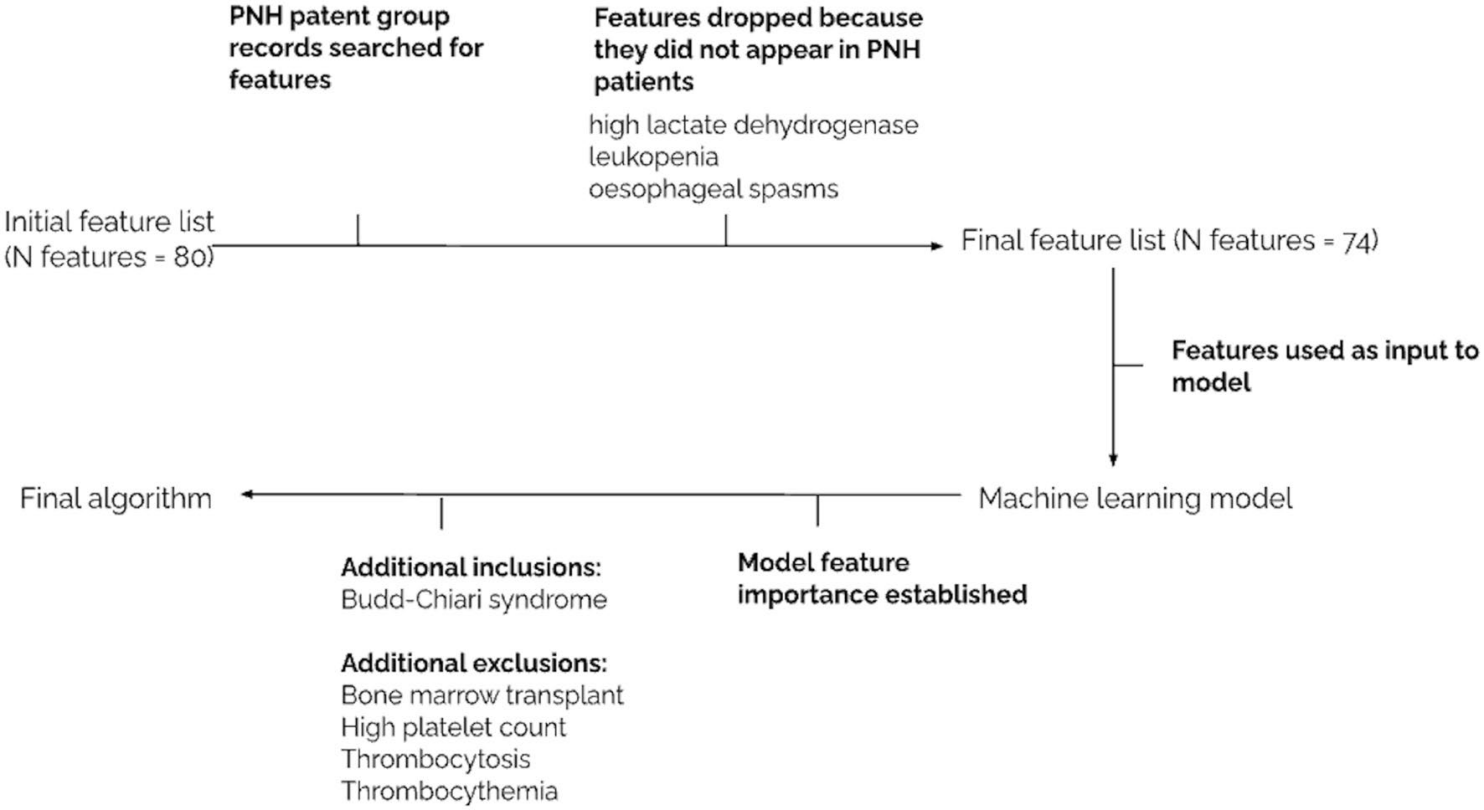
Flowchart demonstrating process for final feature inclusion

Descriptive analysis was conducted on the quantitative dataset to assess suitability as input to a machine learning model. Features that did not appear in this group of PNH patients were dropped from further analysis, as the model would not learn anything useful, these features included: oesophageal spasms, high lactate dehydrogenase and leukopenia (Fig. 1). Furthermore, patients in the PNH group that did not have a single relevant feature in their electronic health record were excluded from further analysis (N = 31).

### Statistical analysis

Chi-Square Test of Proportions was used to test for significant differences in the proportion of each group with each feature. Mann–Whitney U test was used to test for significant differences in the age of onset of each feature between the PNH and control groups.

### Machine learning modelling

An XGBoost classification algorithm was developed; this is a powerful approach which creates an ensemble of decision trees built successively to minimise errors made by previous trees [13]. This classifier is particularly powerful over other classification models such as Random Forest due to its ability to handle incomplete data (e.g. no age of onset data is available where the feature is not present in a record), better performance, flexibility and speed.

To train and test the model, a fivefold nested cross-validation approach was used with stratified bootstrapping applied to each of the 5 outer folds (1000 iterations in total). The benefit of this approach is that it enables: (1) testing of different model parameters to achieve optimal performance for each split, (2) minimising the risk of overfitting the model (learning a lot about seen data but not generalising well to unseen data), (3) computation of confidence intervals to indicate how well the model may perform in different samples, and (4) making use of all PNH patient data for training, which is important for a dataset of electronic health records in a rare disease cohort, where the sample size is modest and features are heterogeneous. Feature importance was approximated using XGBoost’s built-in feature importance method.

### Final algorithm and performance evaluation

**Fig. 2:**
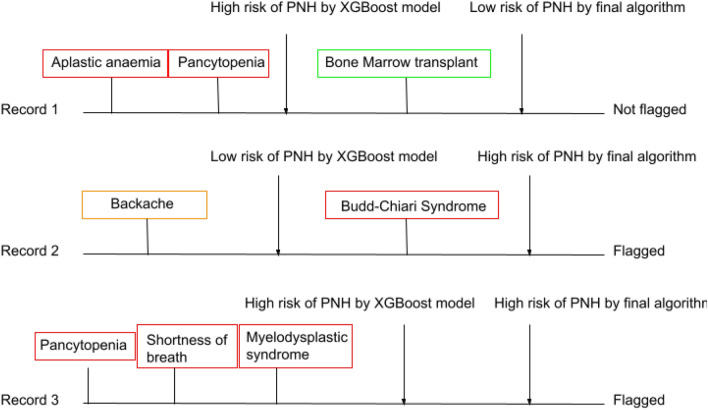
Illustration of how additional exclusion and inclusion criteria could impact final algorithm

To ensure our final algorithm best serves PNH patients and expert clinicians, we took a clinically driven approach both before and after the data-driven machine learning component of algorithm development. With expert guidance, we identified exclusions and flags that could be applied after modelling but before final performance was evaluated (see Figs. 1 and 2). This included excluding patients who had a relevant code which indicated that development of PNH would be highly unlikely and includes: a history of bone marrow transplant, high platelet count, thrombocytosis and thrombocythemia. While these features were used as input into the model for tree-based learning, they were not in the top features of importance and thus it was necessary to also apply these exclusions outside of the model. Finally, the presence of Budd-Chiari syndrome indicates high risk of PNH and clinical guidelines state that anyone with confirmed Budd-Chiari syndrome should be tested for PNH [14], therefore our model includes an additional step which flags any record with Budd-Chiari syndrome, even where the rest of the electronic health record may be sparse or features may not be strong indicators of PNH risk and therefore may not have been considered a positive case by the XGBoost model itself.

Performance of our algorithm was assessed using positive predictive value (PPV) and recall (also known as sensitivity), which are all ideally suited to imbalanced datasets. PPV is understood as “out of the cases that the model classified as positive for the disease, how many had an existing/coded diagnosis for the disease”. Recall is understood as “out of the cases with a coded diagnosis for the disease, how many did the model correctly classify as positive”. In addition, adjusted PPV was calculated. Adjusted PPV recognises that the study data set was enriched with patients with a PNH diagnosis coded in their record and does not represent the true population prevalence. It is important, in this setting, to adjust PPV by prevalence to understand what the PPV could be in the general population. Specificity was also calculated and can be understood as “out of the controls in the study, how many did we correctly classify as controls”. High specificity can be achieved by a model that correctly classifies controls, but poorly classifies cases, as such we consider this a secondary metric and was calculated for reporting purposes only.

## Results

### Sample breakdown

**Fig. 3:**
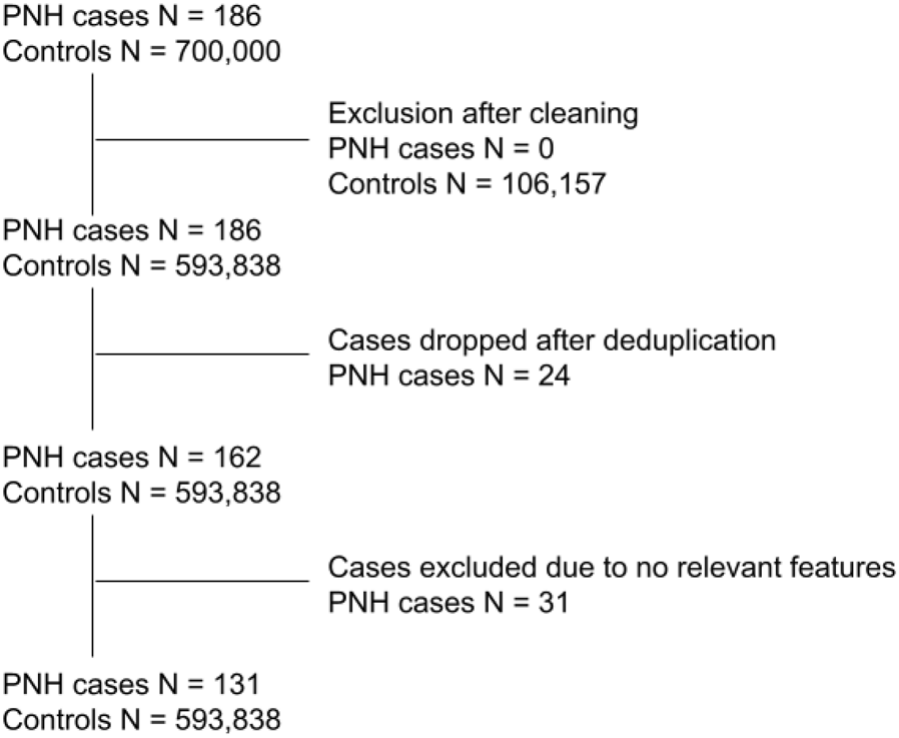
Breakdown of sample after cleaning and preprocessing

186 patients with PNH were identified across OPCRD. Of those 24 were excluded after combining their records with other patient IDs that were deemed to be duplicate records (see Fig. 3). A further 31 were found to have no relevant features in their records and were therefore excluded from further analysis. 700,000 random patient IDs were initially extracted from the whole OPCRD database. Of those, 106,157 were excluded during the cleaning process.

The final sample for modelling consisted of 131 patients in the PNH group and 593,838 controls, yielding a ratio of approximately 1:4533 PNH cases and controls.

131 patients in the PNH group in the OPCRD sample represents a coded prevalence of 5.7 per million. This is lower than the accepted prevalence of PNH in the UK, which is 38.1 per million [1]. This discrepancy could be due to: (1) people with PNH in the OPCRD cohort being underdiagnosed or having their diagnoses unrecorded at the GP level, and (2) the common observation in rare diseases of widely varying prevalence estimates, often caused by methodological differences, sample variations, and the challenges of small patient numbers.

### Sample demographics

At the time of data extract, the whole of the OPCRD sample was made up of 48% males with a mean age of 36.82. The PNH sample was made up of 50% males with a mean diagnosis age of 47.83. The control sample was made up of 48.27% males with a mean index age of 36.82 (see Table 1).

**Table 1:** Basic demographics for the PNH and control groups included in the final XGBoost model

	PNH (N = 131)	Non-PNH (N = 593,838)
Mean index age (std)	47.83 (19.70)	36.82 (24.93)
N Male (%)	79 (50)	285,646 (48.27)

### Clinical characteristics

The clinical characteristics of the cohort can be found in Table 2. In summary, the patients in the PNH group had a significantly higher prevalence of aplastic anaemia, myelodysplastic syndrome, anaemia, haemolytic anaemia, pancytopenia, thrombocytopenia, Budd-Chiari syndrome, other thromboses, embolism, haemoglobinuria, haemolysis, haematuria, abdominal pain, dysphagia, lethargy, backache, urinary tract infections, skin infections, renal dysfunction, shortness of breath, blood transfusions, bone marrow tests, hospitalisations, haptoglobin tests, blood tests and referrals to haematology, gastroenterology, and urology, as compared to the control patients.

**Table 2:** Clinical characteristics of the PNH and control groups included in the final XGBoost model

	Presence of feature N patients (% sample)			Age of onset of feature Median (min, max)		
Feature	PNH	Controls	p-value	PNH	Controls	p-value
Aplastic anaemia	38 (29.006)	33 (0.006)	< 0.0001	32 (8, 82)	25 (2, 89)	0.319
Myelodysplastic syndrome	13 (9.924)	142 (0.024)	< 0.0001	61 (24, 79)	74 (5, 98)	0.005
Anaemia	47 (35.878)	14,950 (2.518)	< 0.0001	46 (14, 82)	46 (0, 100)	0.977
Haemolytic anaemia	12 (9.160)	181 (0.030)	< 0.0001	44.5 (14, 80)	34 (0, 94)	0.12
Pancytopenia	27 (20.611)	148 (0.025)	< 0.0001	53 (14, 79)	60 (1, 93)	0.698
Neutropenia	7 (0.162)	964 (5.344)	0.0084	37 (18, 79)	42 (0, 96)	0.677
Thrombocytopenia	7 (5.344)	432 (0.073)	< 0.0001	50 (23, 79)	46 (0, 93)	0.564
Budd-Chiari syndrome	2 (1.527)	5 (0.001)	< 0.0001	29.5 (27, 32)	31 (25, 76)	1
Thromboses (excluding Budd-Chiari syndrome)	13 (9.924)	11,348 (1.911)	< 0.0001	43 (21, 71)	48 (0, 99)	0.321
Myocardial infarction	2 (1.527)	9107 (1.534)	0.9949	66.5 (64, 69)	62 (0, 99)	0.563
Embolism	7 (5.344)	6933 (1.68)	< 0.0001	57 (21, 83)	67 (0, 100)	0.171
Stroke	4 (3.053)	8643 (1.455)	0.1268	63.5 (48, 83)	69 (0, 100)	0.696
Haemoglobinuria	2 (1.527)	16 (0.003)	< 0.0001	25.5 (25, 26)	48.5 (5, 75)	0.182
Haemolysis	4 (3.053)	2482 (0.418)	< 0.0001	26.5 (16, 43)	51 (0, 98)	0.025
Haematuria	27 (20.611)	12,117 (2.041)	< 0.0001	47 (16, 82)	54 (0, 99)	0.969
Abdominal pain	25 (19.084)	68,004 (11.452)	0.0061	37 (2, 82)	31 (0, 100)	0.208
Dysphagia	3 (2.290)	4171 (0.702)	0.03	74 (48, 78)	59 (0, 99)	0.383
Lethargy, asthenia, fatigue	18 (13.741)	36,873 (6.209)	0.0004	48.5 (21, 79)	40 (0, 100)	0.095
Impotence	5 (3.817)	12,936 (2.178)	0.199	67 (33, 75)	56 (0, 93)	0.21
Backache	42 (32.061)	98,909 (16.656)	< 0.0001	43 (15, 78)	41 (0, 100)	0.289
Proteinuria	1 (0.763)	2369 (0.399)	0.5083	52 (52, 52)	58 (0, 96)	0.773
Urinary related	44 (33.588)	77,260 (13.010)	< 0.0001	43 (11, 82)	39 (0, 100)	0.1
Skin infection	24 (18.321)	50,024 (8.424)	0.0001	47.5 (0, 68)	38 (0, 100)	0.357
Respiratory tract infection	36 (27.481)	127,064 (21.397)	0.0896	46.5 (0, 82)	29 (0, 100)	0.0004
Renal dysfunction	16 (12.214)	25,985 (4.376)	< 0.0001	58 (38, 85)	67 (0, 100)	0.114
Shortness of breath	14 (10.687)	26,035 (4.384)	0.0004	68 (36, 81)	55 (0, 100)	0.032
Blood transfusion	20 (15.267)	1535 (0.259)	< 0.0001	44 (16, 82)	45 (0, 98)	0.92
Bone marrow	6 (4.580)	74 (0.013)	< 0.0001	48 (6, 79)	59.5 (1, 88)	0.476
Hospitalisation	39 (29.771)	46,216 (7.783)	< 0.0001	38 (1, 86)	39 (0, 100)	0.346
Haptoglobin test	4 (3.053)	119 (0.020)	< 0.0001	35.5 (20, 62)	54 (0, 91)	0.35
Blood test	101 (77.099)	300,822 (50.657)	< 0.0001	39 (2, 78)	41 (0, 100)	0.656
Oncology referral	2 (1.527)	3100 (0.522)	0.1107	48.5 (34, 63)	60 (1, 98)	0.416
Haematology referral	37 (28.244)	4146 (0.698)	< 0.0001	51 (18, 84)	54 (0, 98)	0.993
Gastroenterology referral	18 (13.741)	21,423 (3.608)	< 0.0001	49.5 (20, 72)	53 (0, 98)	0.249
Urology referral	16 (12.214)	20,348 (3.427)	< 0.0001	64 (23, 82)	54 (0, 100)	0.066
Bone Marrow transplant	0 (0.000)	63 (0.011)	0.9062	–	29 (0, 80)	–
High platelet count, thrombocytosis, thrombocythaemia	4 (3.053)	7104 (1.196)	0.0506	33 (18, 75)	51 (0, 98)	0.401

### Algorithm performance

**Fig. 4:**
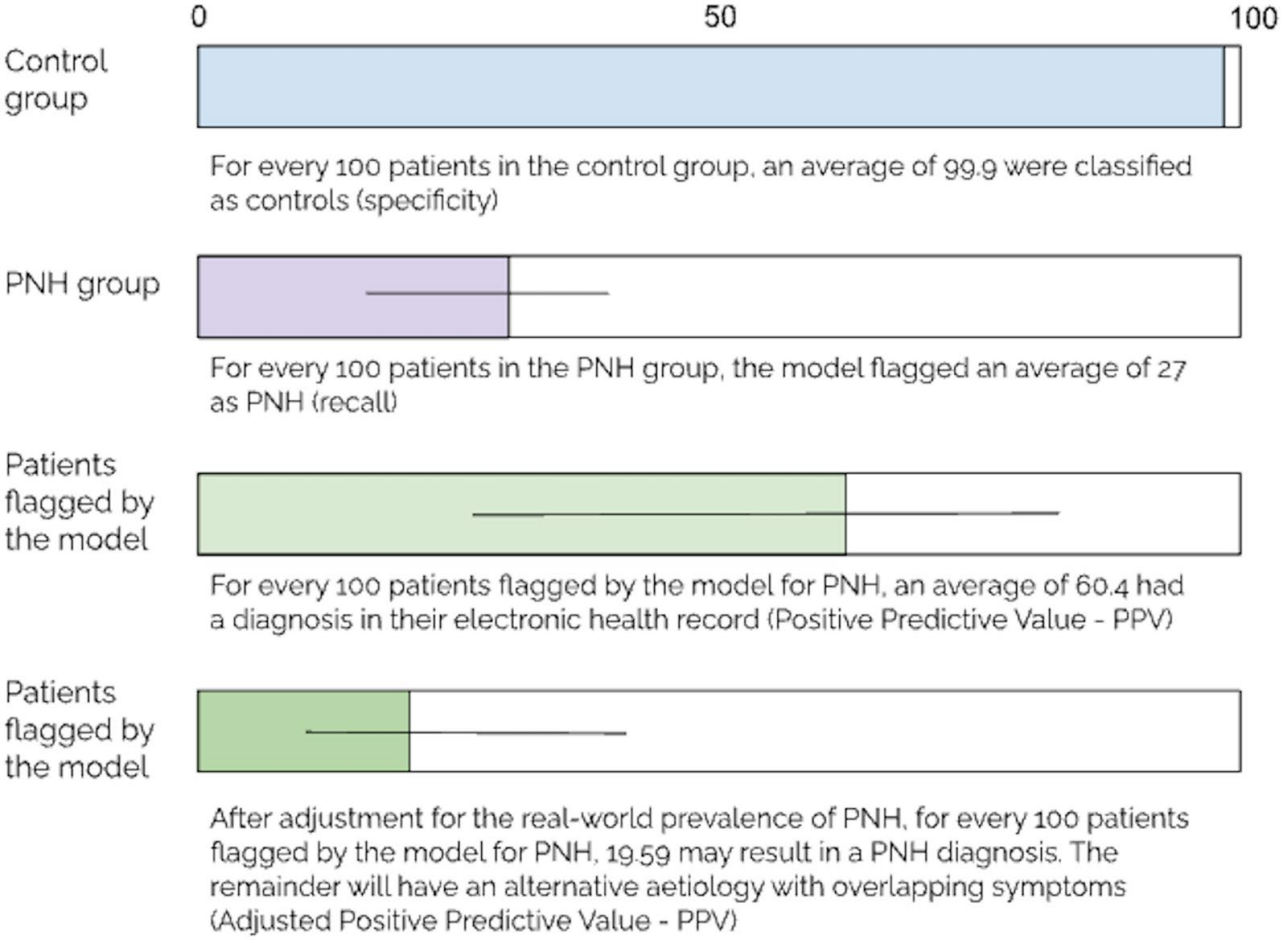
A visual representation of average performance of the algorithm across the 5-folds of cross-validation, including sensitivity, recall, positive predictive value (PPV) and adjusted PPV

A fivefold cross-validation approach was used, a limitation of this approach is that it does not enable reporting of exact numbers of patients flagged or not flagged. However, it does allow for percentages to be averaged across all 5 cross-validation folds, which offers a better representation of performance across different subsets of the dataset; the performance metrics reported here are averages from across all 5 cross-validation folds. Of all the patients in the PNH group, 27% (CI 15–39%) were classified as positive (recall) (see Fig. 4). Out of all the patients classified as positive, 60.4% (CI 33–82%) had an existing diagnosis of PNH coded in their record (PPV). After adjusting for the known rarity of PNH (estimated at 3.81 cases per 100,000 individuals [1]) 19.59% (7.63–41.81) flagged by the algorithm may warrant further investigation for PNH. Out of all the patients in the control group 99.99% were classified as negative (specificity).

**Fig. 5:**
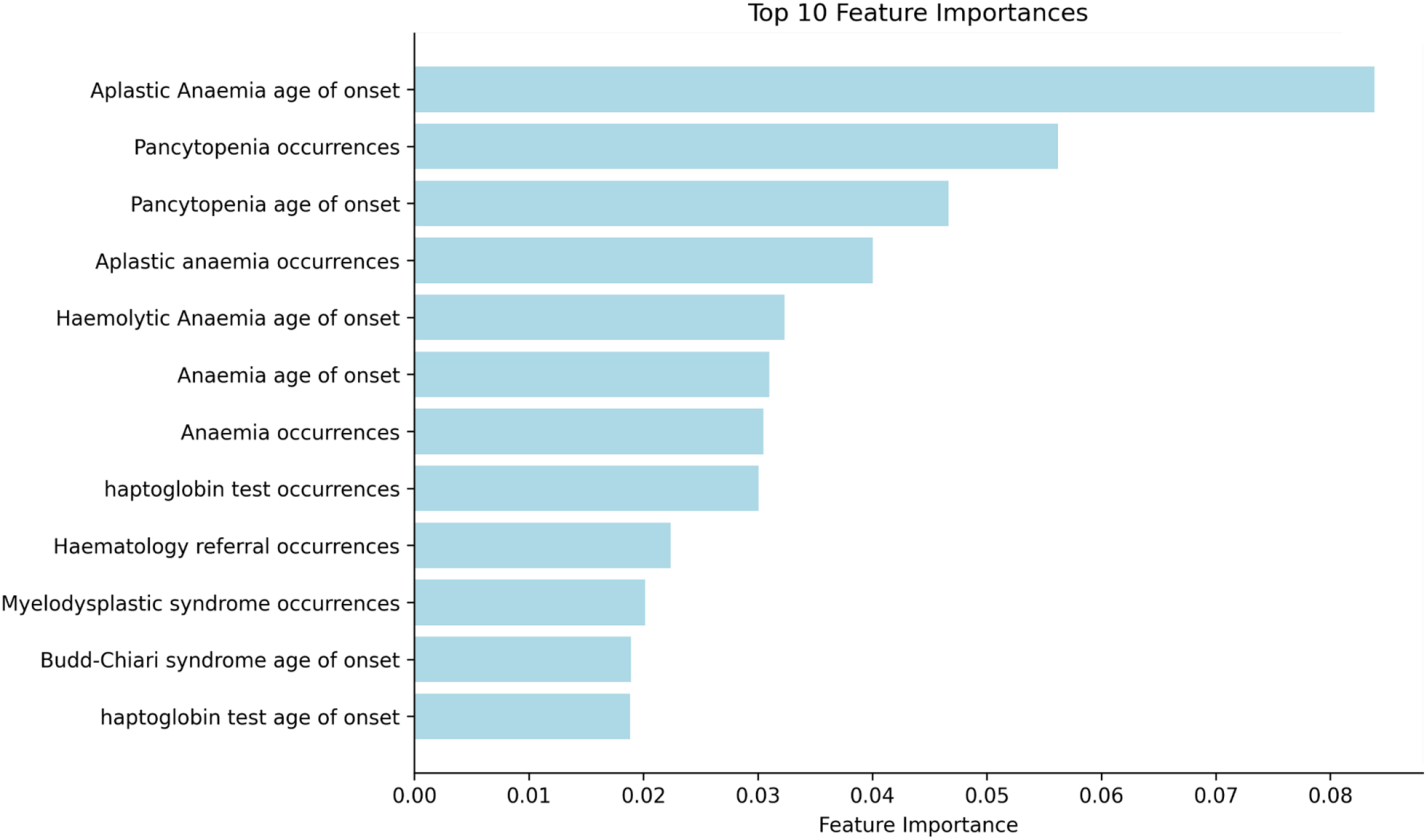
10 features of most importance in the XGBoost model, using XGBoost’s inbuilt feature importance method

The five clinical features of most importance in the model were aplastic anaemia, pancytopenia, haemolytic anaemia, myelodysplastic syndrome and Budd-Chiari syndrome (see Fig. 5). Other features which are detected in patients in the PNH group and contribute as top features in the model are haematology referrals, urology referrals and haptoglobin tests. This means that patients presenting with one or more of these features are most likely to be flagged by the model, as their electronic health records are similar to patients who have already received a diagnosis of PNH.

## Discussion

Tackling long diagnostic delays is a key challenge for all rare diseases, particularly for those where treatments which can reduce morbidity and mortality are available, such as Paroxysmal Nocturnal Haemoglobinuria (PNH). Thirty-five percent of PNH patients in the UK report symptoms at least twelve months before receiving a diagnosis and 13% receive at least one misdiagnosis [4]. In the present data, roughly 80% of PNH patients have relevant features coded in the structured primary care electronic health record before their diagnosis and could potentially be diagnosed earlier. This was a retrospective study, exploring whether a model could be built to identify undiagnosed PNH patients (using features that occurred prior to the diagnosis) from their electronic health records. We found that 60.4% of patients flagged by the algorithm had an existing diagnosis of PNH coded in their electronic health record and of all the patients included in the model with a PNH diagnosis coded, 27% were flagged. After adjustment for the real-world prevalence of PNH, approximately 1 in 5 patients flagged by the algorithm could have PNH. This indicates that such a tool, if deployed in the real world, may help in reducing diagnostic delays for these patients.

The study has a number of strengths. The first is the combination of a clinically led and data-driven approach. The initial feature list was developed in consultation with a leading Consultant Haematologist at the Leeds National PNH centre, one of the two PNH centres in the UK. Furthermore, as the machine learning model was developed, patient records were reviewed with this specialist and features refined to achieve optimal performance. By combining an additional rules-based approach, in addition to the machine learning model, we reduce false positives by excluding those with low risk (bone marrow transplant in record). We also maximise the number of appropriate patients that are flagged for the disease by including additional flags for Budd-Chiari syndrome.

This study demonstrates the potential for improving PNH diagnosis through an algorithm utilising structured primary care electronic health record data. Despite the inherent limitations of structured data – sparsity, coding errors, and lack of granularity – we found that 27% of patients with PNH diagnosis coded in their electronic health record could be flagged based on clinical features predating the diagnostic code. While this may seem modest, it is a promising result given the ultra-rare nature of PNH. Given the need to reduce diagnostic waiting times, misdiagnosis and number of referrals, further investigation into the validity and clinical utility of this algorithm is warranted. If successfully validated and implemented, this algorithm could improve outcomes for patients with PNH.

The study has several limitations. Firstly, an assumption has been made that the presence or absence of a diagnosis code for PNH indicates the true status of the patient. This may not always be the case, for example, a diagnosis may have been made by a specialist and this may not have been recorded in the primary care electronic health record. It is also possible that where codes for PNH do exist, they may be inaccurate and actually reflect a family history, suspicion of the disease or a coding error. We accept and in fact assume, given the real-world nature of the data, that there may be PNH patients in the control sample we have trained the algorithm on and this limits the predictive power of the algorithm and impacts the confidence we have in performance metrics. With future access to ground truth data on PNH diagnosis status, we may see improvements in algorithm performance.

Other factors that impact real-world electronic health data include tracking patients records as they move practices, for example, some patient’s records may become split between two different practices and assigned different unique IDs. This may result in partial records, where PNH patients seem to have fewer or no relevant clinical features, while a patient who appears to be a control (with no coded diagnosis for PNH in the electronic health record) may have many relevant features. Furthermore, the algorithm has been trained on a sample of patients with a diagnostic SNOMED-CT code for PNH and that have at least one relevant feature coded in primary care electronic health record. As a result, cases where relevant symptoms have appeared in the individual, but have not been recorded in the electronic health record, will not be flagged by the algorithm. Finally, high lactate dehydrogenase is considered clinically to be a very good marker of untreated PNH, however it is not well coded in primary care electronic health records and thus was not included in the model. This highlights the importance of primary care electronic health coding systems being utilised regularly and for test results conducted in specialist settings to be passed on to GP’s, to enable tools such as this to facilitate practitioners and end the diagnostic odyssey for rare disease patients.

Future directions for this research include validating the model in an independent dataset to assess its generalizability and real-world performance. While the cross-validation approach used in this study provides valuable insights, external validation is important for confirming the model's ability to accurately identify potential PNH patients in different populations and healthcare settings. Furthermore, a follow-up investigation of the patients in the control group flagged by our model could help determine if any truly represent undiagnosed or, diagnosed but not coded, PNH cases, further validating the tool's effectiveness in identifying potential patients. Additionally, incorporating unstructured data from electronic health records, such as clinical notes and discharge letters, could enhance the model's performance by capturing more nuanced medical details. Finally, an assessment of the impact of our tool on reducing the diagnostic delay for PNH patients would be beneficial. This could involve quantifying the time difference between initial symptom presentation and diagnosis for patients identified through this method compared to conventional pathways.

## Conclusion

In conclusion, we demonstrate that structured data captured in UK primary care electronic health records can be leveraged to develop a case-finding algorithm for PNH. In this retrospective study, we combined clinical understanding of the presentation of PNH with machine learning to evaluate how well an algorithm could identify PNH whose health data is consistent with patterns observed in diagnosed PNH cases. Our results show that for every five cases flagged by the algorithm, one case could be a PNH patient. Further work is needed to validate and assess performance in independent samples, with the ultimate goal being real-world deployment. If successful, this tool has the potential to reduce diagnostic delays for PNH patients.

## Supplementary Information

Supplementary Material 1.

## Data Availability

The data that support the findings of this study are available from Optimum Patient Care. Restrictions apply to the availability of these data, which were used under licence for the current study, and so are not publicly available.

## Abbreviations

PNH: Paroxysmal Nocturnal Haemoglobinuria
OPCRD: Optimum Patient Care Research Database
GP: General Practitioner
PPV: Positive predictive value
UK: United Kingdom
CTV3: Clinical Terms Version 3
SQL: Structured Query Language
ID: Identity
NHS TRUD: National Health Service Technology Reference Update Distribution
