# Supplementary material for: A machine learning algorithm for the detection of paroxysmal nocturnal haemoglobinuria (PNH) in UK primary care electronic health records
Source: Orphanet J Rare Dis. 2024 Oct 13;19:378. doi: 10.1186/s13023-024-03406-4 (PMC11479535; doi:10.1186/s13023-024-03406-4)
Supplement: Supplementary file 1 — Supplementary Material 1. [file 13023_2024_3406_MOESM1_ESM.docx]

| **Supplementary Table 1:** Basis for feature selection for each feature included in the model | | | | |
| --- | --- | --- | --- | --- |
| Feature | Literature | Expert Clinical Insight | Case Reports | Patient electronic health record |
| Aplastic Anaemia |  |  |  |  |
| Myelodysplastic syndrome |  |  |  |  |
| Anaemia |  |  |  |  |
| Haemolytic Anaemia |  |  |  |  |
| Pancytopenia |  |  |  |  |
| Neutropenia |  |  |  |  |
| Thrombocytopenia |  |  |  |  |
| Budd-Chiari Syndrome |  |  |  |  |
| Thromboses (excluding Budd-Chiari) |  |  |  |  |
| Myocardial Infarction |  |  |  |  |
| Embolism |  |  |  |  |
| Stroke |  |  |  |  |
| Haemoglobinuria |  |  |  |  |
| Haemolysis |  |  |  |  |
| Haematuria |  |  |  |  |
| Abdominal pain |  |  |  |  |
| Dysphagia |  |  |  |  |
| Lethargy, asthenia, fatigue |  |  |  |  |
| Impotence |  |  |  |  |
| Backache |  |  |  |  |
| Proteinuria |  |  |  |  |
| Urinary Tract Infection |  |  |  |  |
| Skin Infection |  |  |  |  |
| Respiratory Tract Infection |  |  |  |  |
| Renal dysfunction |  |  |  |  |
| Shortness of breath |  |  |  |  |
| Blood transfusion |  |  |  |  |
| Bone Marrow |  |  |  |  |
| Hospitalisation |  |  |  |  |
| Haptoglobin Test |  |  |  |  |
| Blood Test |  |  |  |  |
| Oncology referral |  |  |  |  |
| Haematology referral |  |  |  |  |
| Gastroenterology referral |  |  |  |  |
| Urology referral |  |  |  |  |
| Bone Marrow transplant |  |  |  |  |
| High platelet count, Thrombocytosis, Thrombocythaemia |  |  |  |  |
